# Genetic evaluation of health costs in US organic Holstein calves and cows

**DOI:** 10.3168/jdsc.2023-0377

**Published:** 2023-08-19

**Authors:** L.C. Hardie, I.W. Haagen, B.J. Heins, C.D. Dechow

**Affiliations:** 1 Department of Animal Science, Pennsylvania State University, University Park, PA 16802; 2 Department of Animal Science, University of Minnesota, St. Paul, MN 55108

## Abstract

Minimizing the incidence of disease on organic dairy farms is important for both economic and animal welfare purposes. The objective of this study was to estimate genetic parameters for total disease treatment costs using producer-recorded treatments in organic Holstein dairy calves and cows. Individual cow and calf health data were collected from 16 USDA certified organic farms from across the United States. Eleven of these farms provided treatment costs for some or all of the following cow health issues (mean cost): mastitis ($46.10), milk fever ($39.05), ketosis ($29.81), metritis ($28.66), retained placenta ($45.59), displaced abomasum ($439.71), lameness ($66.36), indigestion ($22.94), respiratory ($48.35), and died ($64.98). These farms also provided the following health costs for calves (mean cost): respiratory ($56.37) and scours ($25.21). Costs included consultant fees, therapeutics, and producer labor. The total lactational health cost (HCOST) was analyzed using animal models adjusted for the fixed effects of lactation and herd and the random effect of herd-year-season of calving with animal relationships based on the blending of pedigree and genomic relationships established from 2,347 genotyped cows. Along with HCOST, the binary traits stayability and presence of disease were included in a trivariate model such that lactations absent of disease were considered to be missing HCOST. To estimate the genetic relationship between nulliparous and primiparous health costs, a 2-trait linear model was fitted for total nulliparous health costs (NHCOST) and first lactation HCOST. The most expensive cow-lactation was $643.86 and 26.5% of lactations encountered disease. The heritability for HCOST was 0.03 ± 0.01, and the repeatability was 0.21 ± 0.01. The heritability of NHCOST was 0.06 ± 0.01, and the genetic correlation between NHCOST and HCOST was 0.98 ± 0.51. Traits representing the repeated nature of disease have a genetic component that should foster improved disease resistance among organic Holstein dairy cows. However, total cost of disease did not lead to gains in genetic variation over consideration of disease traits considered as binary variables and is a more laborious phenotype to obtain, diminishing its appeal for use in routine genetic evaluations.

## Highlights

• Costs to treat disease on US organic farms have not been well characterized and widely vary among these farms.
• Cost to treat disease can be considered a trait of a calf or cow and is lowly heritable.
• There is a genetic relationship between cost to treat disease in a calf and a cow.

## Graphical Abstract

**Figure:**
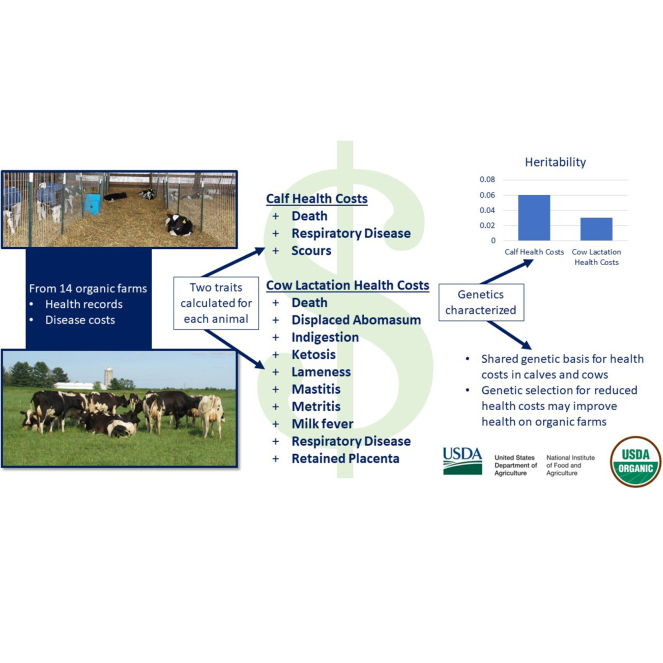
**Summary:** Minimizing the incidence of disease can be achieved through genetic selection and is important on organic dairy farms for both economic and animal welfare purposes. Our aim was to characterize the genetics of health costs on US organic farms. We combined health data from 14 organic farms with average treatment costs from 11 of these farms to estimate the health costs for each calf and lactations of each cow. The following health issues were considered: death, displaced abomasum, indigestion, ketosis, lameness, mastitis, metritis, milk fever, respiratory disease, retained placenta, and scours. Health costs were lowly heritable in both calves and cows (0.06 and 0.03) but related over the lifetime with calves genetically prone to incurring higher health costs also more likely to incur higher health costs as cows. Genetic selection for disease resistance will reduce health costs among organic Holstein dairy cattle.

Improving the health of organic dairy cattle is critical for animal welfare and economic sustainability of organic dairy farms. Genetic improvement of disease resistance is one potential solution, and national genetic evaluations for common health traits in dairy calves and cows are now available but are often based on the binary incidence of the disease (**BINSICK**) during a lactation (Vukasinovic et al., 2017; Gonzalez-Peña et al., 2019; Parker Gaddis et al., 2020). For traits with repeated incidence during a lactation or youngstock rearing period (e.g., mastitis or scours), these models suppress the amount of genetic variation that is captured (Chang et al., 2004), and strategies that capture the recurring nature of disease may offer greater opportunity to improve disease resistance. Models that evaluate count data have been shown to more accurately identify diseased animals and predict mastitis costs in primarily conventional cows (Vazquez et al., 2009). Combining count data with treatment costs, total lactational health costs have been shown to be more heritable than the binary presence or absence of a disease during a lactation (Donnelly, 2017).

Organic dairy cattle in the United States are not permitted to receive antibiotics and keep their organic status (USDA-AMS, 2020), eliminating many therapeutics commonly used on conventional dairy farms (Pol and Ruegg, 2007). Alternatively, organic dairy farmers may turn to therapeutics such as tinctures of plant extracts (e.g., garlic), homeopathy, vitamins, and essential oils (Pol and Ruegg, 2007; Brock et al., 2021). Such methods demonstrate wide variation in cost per dose and time to administer and, therefore, in comparison to conventional farms, may lead to differences in selection pressure received by traits.

The objectives of this study were to determine disease costs for organic producers and to use producer-reported disease treatment costs and disease incidence to estimate genetic parameters for total lactational health costs, nulliparous health costs, and the genetic relationship between nulliparous and primiparous health costs for US organic Holsteins. We hypothesize that traits that reflect the repeated nature of disease will capture more genetic variation than consideration of the disease as a binary trait.

Approval for this research was granted under Pennsylvania State Institutional Animal Care and Use Committee protocol #47560. Data were collected from 16 USDA certified organic herds recommended by industry personnel for keeping thorough records and grouped into 3 regions: East (n = 4), Midwest (n = 6), and West (n = 6). Disease incidence records, cow lifespan, and pedigree records were obtained by retrieval of herd software (DairyComp 305, DHI-Plus, PCDart) backups or copy of paper records during herd visits by research personnel. More details related to cow traits are provided in Hardie et al. (2021, 2022) and for youngstock traits in Haagen et al. (2021). For inclusion in analyses, animals must have been born after one year before the herd receiving organic certification such that the entirety of the animal's lactating years occurred under organic management. For each herd, only events occurring in the same year or after the first recorded case of each disease were considered eligible for that disease. Herds with less than 1% of eligible nulliparous animals or cow lactations recording a case of a given disease were excluded from analyses for that disease.

All participating herds were invited to provide disease treatment costs. Costs were received from 11 herds, with all regions represented, either by filling out and returning a worksheet provided by research personnel (n = 5) or by verbally reporting specific therapeutics and their costs to research personnel completing the worksheet (n = 6). Producers did not need to provide treatments; for each disease, they were simply asked to provide the cost of consultant treatment costs (e.g., veterinary, hoof trimmer, or rendering truck) and the costs associated with farmer-administered treatments. For consultant treatments, we asked the producer to fill in the following 3 fields of data: (1) an estimate of the percentage of cows recorded with the disease that received consultant care, (2) whether this consultant care was part of a routine visit or one-time visits, and (3) a total cost that included consultant time and supply costs. For routine visits such as routine hoof trimming, “stop charges” were divided among an estimate of the number of animals trimmed at each appointment, whereas for special visits, such as emergency veterinarian visits, the entire stop charge was applied to the disease cost.

For each farmer-administered treatment, we asked for the following 4 fields of data: (1) an estimate of the percentage of cows recorded with the disease that that received that treatment, (2) the total cost of therapies administered, (3) the amount of time per day spent by the producer treating the cow, and (4) the number of days the treatment was administered. We applied the rate of $18.10 per hour for producer time, the quotient of the annual average weekly wage ($724) for dairy cattle and milk production employment, divided by 40 h per week (DOL-BLS, 2019). Farms were able to provide these data for up to 3 different treatments for each disease, and most often, when multiple costs were provided, one cost was provided for mild and one cost for severe cases of disease. For each disease, we weighted the cost of each treatment (including consultant charges, on-farm treatment costs, and producer time) by the frequency of its application and summed across the weighted costs to get a total farm treatment cost for the disease. Descriptive statistics for treatment costs across farms are provided in Table 1.

**Table 1:** Descriptive statistics of producer-supplied organic disease treatment costs

Disease^1^	Cost ($)						Farms supplying costs (n)	Threshold for new case^4^
	Consultant^2^	Therapeutic^2^	Producer time^3^	Total: median	Total: mean	SD		
Cow								
DA	279.17	102.12	58.42	485.68	439.71	139.95	3	8 d
Died	57.38	0.00	10.94	80.37	64.98	43.43	7	1 per life
Indigestion	0.00	12.01	10.94	19.57	22.94	8.65	7	5 d
Ketosis	0.21	13.97	15.63	26.91	29.81	13.43	7	5 d
Lameness	6.88	6.17	4.80	41.08	66.36	11.84	10	2 d
Mastitis	4.67	19.59	19.66	19.48	46.10	76.34	10	7 d
Metritis	6.79	11.97	9.90	23.58	28.66	16.17	7	3 d
Milk fever	19.86	12.03	7.15	21.17	39.05	37.73	11	2 d
Respiratory	21.25	16.92	10.18	39.91	48.35	39.65	4	3 d
Retained placenta	3.95	25.98	15.66	28.14	45.59	56.33	8	1 per lact
Calf^5^								
Respiratory	10.94	24.29	21.14	27.80	56.37	81.67	8	5
Scours	2.29	12.24	10.69	25.36	25.21	12.58	10	4

1 DA = displaced abomasum; DA cost not weighted by frequency used on farm.

2 Consultant and therapeutic costs were mean costs.

3 Cost per hour used was $18.1.

4 lact = lactation; treatment costs for retained placenta were applied at most once per lactation; costs for dying on farm were applied at most once per life; listed thresholds for lameness and mastitis refer to minimum time required to assign a new case on the same hoof or quarter or reapply the treatment cost, but new cases were assigned to different hooves or quarters at any time.

5 Calf “died” costs were assigned at $6.00.

For each cow lactation, the count of cases of each disease was determined and multiplied by the respective mean treatment cost for that disease, followed by taking the sum of these products to get the total lactational health cost (**HCOST**) for that disease. For nulliparous animals, the count of cases was multiplied by the respective mean treatment cost for each disease and summed across disease type until 18 mo of age to calculate total nulliparous health costs (**NHCOST**). A waiting period for declaring a new case of disease was determined based on the farm-reported treatment length. Specifically, to define the waiting periods, we calculated the average across farms of the maximum product of treatment length (d) multiplied by frequency of treatment administration. However, for mastitis and lameness, within the waiting period, a new case was declared if a different hoof or quarter was recorded as diseased. For these cases, the treatment cost was only applied to cases meeting the waiting period because most of the treatments reported by organic farmers in previous studies are oral or parenteral (rather than intramammary, for example), and thus are likely not doubled in administration (Pol and Ruegg, 2007; Brock et al., 2021). Figure 1 depicts the proportion of cases attributed to each disease for cows. Herd-years not recording mastitis were ineligible for the trait HCOST. In addition, a herd had to record both respiratory disease and scours for an animal to receive NHCOST.

**Figure 1:**
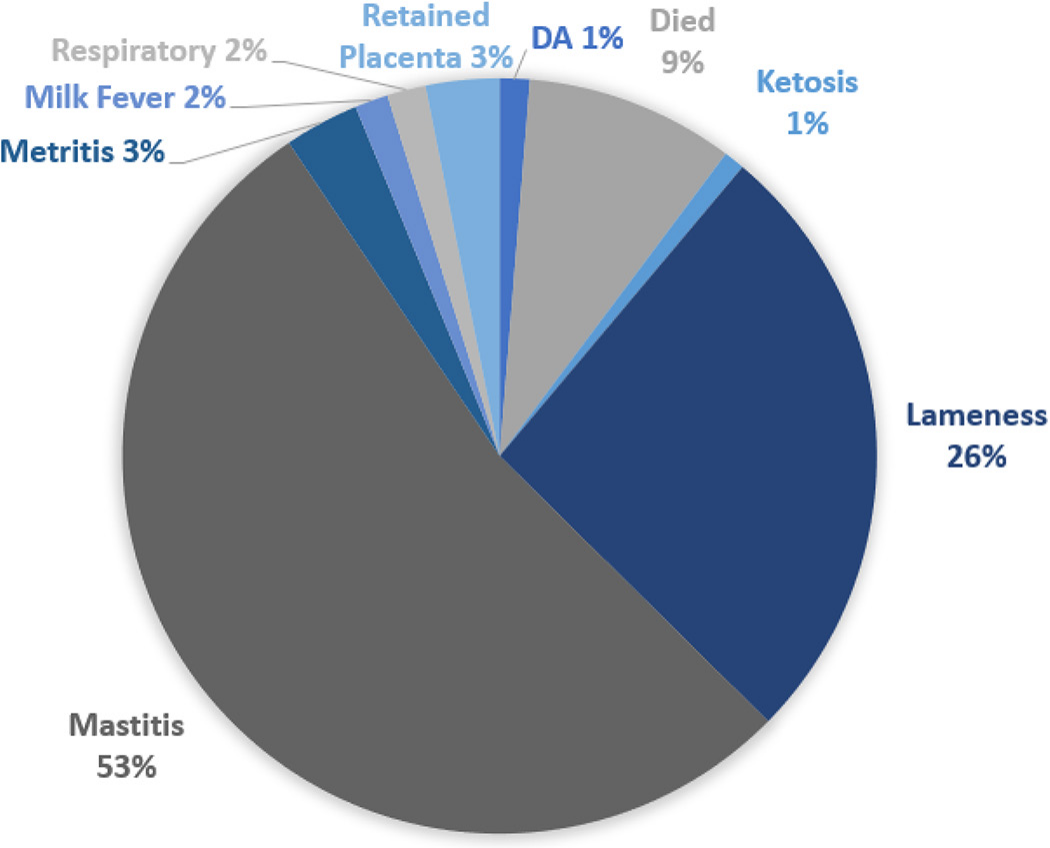
Breakdown of total recorded health events. DA = displaced abomasum.

Stayability is a binary variable (1 = stayed, 0 = departed) reflecting the completion of a given period of time and can be used to reflect longevity (Hardie et al., 2021). We devised stayability phenotypes through a given lactation because as a lifelong measured trait, it should mitigate the impact of selection bias on traits measured later in life that are correlated with traits on which culling decisions are made earlier in life. Stayability was considered in trivariate analyses for cost traits.

Animal relationships were established by pedigree, genotypes, or both, and more details are available about the pedigree (Hardie et al., 2021) and genotypes (Hardie et al., 2022). Phenotyped animals were required to have a Holstein sire and 3 generations of Holstein or unknown breeds for maternal lineage. A total of 2,347 cows with greater that 87.5% Holstein breed-based representation were genotyped using commercially available SNP chips. Genotypes were imputed to 80k and provided by the Council on Dairy Cattle Breeding.

We were interested in genetic parameters for the 2 health traits HCOST and NHCOST. The following linear predictor was used for HCOST:

w_ijkl_ = μ + lact_i_ + herd_j_ + a_k_ + pe_k_ + hysc_l_,

where w_ijkl_ is a function of the expected HCOST, μ is the intercept, lact_i_ is the fixed class effect of lactation (5 levels), herd_j_ is the fixed class effect of herd j (16 levels), a_k_ is the random effect of animal k, pe_k_ is the permanent environment effect for animal k, and hysc_l_ is the random effect of herd-years-season of calving l (465 levels). Random effects were assumed to follow a multivariate normal distribution

$$\left[\begin{array}{c} a\\ \begin{array}{c} pe\\ hysc \end{array} \end{array}\right]N\left[\begin{array}{cc} 0, & \left[\begin{array}{ccc} H\sigma_{a}^{2} & 0 & 0\\ 0 & I\sigma_{pe}^{2} & 0\\ 0 & 0 & I\sigma_{hysc}^{2} \end{array}\right] \end{array}\right],$$

where **a**, **pe**, and **hysc** are the vectors containing the animal, permanent environment, and herd-year-season of calving effects, respectively; **H** is a genetic relationship matrix with variance $\sigma_{a}^{2};$ **I** is the identity matrix; $\sigma_{pe}^{2}$ is the permanent environmental variance; and $\sigma_{hysc}^{2}$ is the variance among herd-year-seasons of calving. The **H** matrix combines pedigree and genomic information to establish animal relationships (Aguilar et al., 2011).

The cost traits were evaluated using trivariate mixed models that included stayability, BINSICK, and HCOST. The linear predictor used for HCOST was used for BINSICK, and with one modification, also for stayability. For stayability, the herd-year-season of birth replaced the herd-year-season of calving, accommodating lactations with missing calving dates. These analyses were performed using the software THRGIBBS1F90 as part of the BLUPF90 family of programs (Misztal et al., 2018a). Nonzero covariances were assumed among the traits for the same random effect but not across effects. Residuals for HCOST were expected to be independent and normally distributed but each with its own variance $\sigma_{e}^{2}.$ $\sigma_{e}^{2}.$ $\sigma_{e}^{2}.$ $\sigma_{e}^{2}.$ These traits followed an identity link to their predictors. We chose to evaluate HCOST only among diseased animals (BINSICK = 1) so that the variance of health costs was not overshadowed by the absence of cost for the many nondiseased animals. Because of the binary nature of stayability and BINSICK, variation for these traits is described as an underlying random variable, liability, whereby if the liability is greater than 0, the outcome is stayed or diseased, respectively. For these 2 traits, residuals were also assumed to be independent and normally distributed with mean of 0 and variances set to 1, following the probit scale. Stayability and BINSICK were associated with their linear predictors via the inverse normal cumulative density function.

To account for differences for opportunity for disease, lactation length was tested as a fixed effect for HCOST, but model fit did not improve according to deviance information criterion (DIC) values and was omitted for all analyses (Masuda, 2019). For all traits, variances for random systematic effects were excluded in the calculation of heritability and repeatability.

Nulliparous health costs were evaluated in a bivariate mixed model with first lactation HCOST using AIREMLF90 (Misztal et al., 2018b). Predictor variables remained the same for HCOST as previously described; for NHCOST, fixed effects were adjusted to include intercept, the class effect of year-season of birth, dam age (in years), and opportunity age in days until 18 mo of age, and random effects included herd-year-season of birth, additive genetics, and random residual. To reduce selection bias, animals were required to have a nulliparous health cost in order for their first lactation record to be included in the bivariate model.

Of the 35,667 lactations in this study, 26.5% incurred disease. A more thorough discussion on the incidence rate for disease in this data set can be found in Hardie et al. (2022), but in brief, it was suggested that sensitivity of disease definition, herd lactational incidence criteria for inclusion in the data set, and evidence that organic herds have lower incidence of disease could contribute to a lower than expected disease incidence. The maximum number of cases across all diseases during a lactation was 14. For nulliparous animals, 39.7% of animals incurred no disease. Scours was the most prevalent nulliparous disease and was highly variable with herd incidence ranging from 4.5% to 59.6% and a median herd incidence of 18.0%. These incidence rates and the reported health costs cannot be considered representative of the US organic dairy industry because farms were not randomly selected.

The number of farms supplying health costs varied widely depending on the trait, ranging from 3 (displaced abomasum; **DA**) to 11 (milk fever). Multiple farms reported that upon switching to organic or feeding strategies that meet the Grass-Fed labeling requirements (https://organicplustrust.com/), or both, their incidence of DA had drastically declined or vanished; therefore, they did not have organic treatment values readily available to report or no DA treatment costs to report at all. These testimonies indicating decline in DA incidence are supported by those reported by Ohio organic dairy farmers in Brock et al. (2021). We did not specifically include on the worksheet fields for indigestion/off-feed (7 responses) or respiratory disease in cows (4 responses), so these values were only provided as other costs either due to the farmer's intuition or by verbal prompt from the researcher recording costs during herd visits, likely explaining their limited number of responses. We also did not include a field for calf death costs, so we estimated this cost based on observed practices across farms and in relation to cow death costs at $6.00.

Overall, the number of days we used to declare new cases of disease matched those previously used for genetic analyses of disease count and costs. Hazel et al. (2020) used a 3-d minimum for repeat cases on the same hoof and 5 d for repeat mastitis cases on the same quarter. However, they reapplied the cost when a new hoof or quarter was diseased, regardless of the time thresholds. Vallimont et al. (2009) applied 7-d and Vazquez et al. (2009) applied 6-d minimums for declaration of a new case of mastitis, not providing comment regarding if these were quarter-specific minimums. Hazel et al. (2020) generally applied a 5-d minimum to metabolic diseases, which matched our ketosis and indigestion thresholds, and they limited retained placenta (as we did) and DA incidence to once per lactation.

Our health trait costs varied widely in their alignment with those previously reported for national data sets primarily based on conventional cows. Among our respondents, mean treatment costs were generally greater than median costs with the greatest proportional difference occurring for mastitis for which the median ($19.48) was only 42.3% of the mean ($46.10). When mean treatment cost differed substantially from median cost, we reevaluated costs with the producers to ensure accuracy. Because we were confident that the costs provided by producers were accurate, we chose to continue with the mean and assumed it reflected a wider range of herds than the median in our data. Costs for a case of mastitis previously used in genetic analyses ranged from $72 to $211 (Vazquez et al., 2009; McNeel et al., 2017; Parker Gaddis et al., 2020); however, these studies considered varying levels of costs for discarded milk and decreased milk production for the remainder of the lactation. Dubrovsky et al. (2020) estimated the cost for first treatment of respiratory disease to be $36.46 in conventional preweaning calves. While lower than our estimate in nulliparous animals, we also included events occurring until 18 mo of age, which likely influenced dosage and labor costs.

We chose not to consider a cost for discarded milk primarily for 2 reasons. First, because most of the treatments used by organic farmers that do not require loss of organic status also do not have required milk withholding periods; for most cases of disease, milk would not need to be withheld. In cases of clinical mastitis whereby milk would be nonsalable because of abnormal properties, milk would need to be withheld from the bulk tank. However, from a survey of Canadian dairy farmers, the median duration of discarding milk for cases of mastitis when treatments were not administered was 2 d and 6 d when clinical mastitis was treated, which included drug withdrawal time (Aghamohammadi et al., 2018). Second, we assumed that milk withheld from the bulk tank would be fed to calves, thereby earning value through calf growth. While other authors impose a cost for discarded milk despite the assumption of feeding it to calves (McArt et al., 2015), feeding organic milk replacer is more expensive versus feeding whole organic milk, thus producers spare themselves this added expense by feeding discarded milk (Sharpe and Heins, 2021).

Our goal was to simply evaluate total health costs as a direct outcome of the episode of the disease and use it as a proxy for disease resistance rather than a thorough cost analysis that included implications on production, reproduction, and longevity traits. Therefore, we did not consider downstream costs such as long-term reductions in milk production, extended days open, or increased susceptibility to culling. Consequent of this goal, in one herd where most cows with DA were sold rather than treated, we weighted the supplied DA treatment cost by 100% rather than by the proportion of cases receiving the treatment as done for all other farms and all other traits. Through this approach, we could better gauge what treating a DA would cost an organic farmer. Time associated with record-keeping was not considered in our study, although this was a reported cost for organic producers surveyed in Brock et al. (2021).

Heritability of HCOST was 0.03 ± 0.01 and is provided in Table 2. This estimate is comparable to the binary estimate for all health events (0.05 ± 0.01) from the same data set (Hardie et al., 2022). Repeatability was low to moderate with an estimate of 0.21 ± 0.02. While HCOST was not normally distributed, log-transformation did not improve heritability and provides less intuitive results so we focused on HCOST. Our heritability estimate for NHCOST was 0.06 ± 0.01. This was slightly lower than heritability estimates of binary disease traits in nulliparous animals such as respiratory disease and scours in a similar population where heritabilities were 0.10 and 0.08 for respiratory disease and scours, respectively (Haagen et al., 2021). Our hypothesis was that these health cost traits that reflect the repeated nature of disease will capture more genetic variation than consideration of the disease as a binary trait. Our heritability estimates do not support this hypothesis, perhaps due to the composite nature of the health costs traits. We estimated a genetic of correlation 0.98 ± 0.51 between NHCOST and first lactation HCOST that, albeit with a large standard error, suggests that increased disease costs during the rearing period are genetically correlated with disease costs during first lactation.

**Table 2:** Estimates of genetic parameters for multiple-incidence health cost traits

Trait	Total lactations	Max^1^ ($)	Genetic parameter		
			Genetic variance	Heritability	Repeatability
Lactational health costs	35,667	643.86	168.74	0.03 ± 0.01	0.21 ± 0.02
Nulliparous health costs^2^	15,082	307.11	67.70	0.06 ± 0.01	

1 Maximum lactational cost for health costs.

2 For nulliparous health costs, total lactations represent the number of individuals.

To our knowledge, this is the first study reporting treatment costs on US organic farms and their use in the genetic evaluation of health costs in US organic dairy cattle. We have demonstrated genetic variation in disease costs among organic animals such that they may be valuable for the genetic improvement of organic dairy cattle. However, by considering total cost of disease, gains in genetic variation over disease traits considered as binary variables were nonexistent and acquiring cost phenotypes is much more laborious to obtain, diminishing the appeal to using these traits routinely in genetic evaluations.
